# Product Lifecycle Management as Data Repository for Manufacturing Problem Solving

**DOI:** 10.3390/ma11081469

**Published:** 2018-08-18

**Authors:** Alvaro Camarillo, José Ríos, Klaus-Dieter Althoff

**Affiliations:** 1 Exide Technologies SAS, 5 allée des Pierres Mayettes, 92636 Gennevilliers, France; 2 Mechanical Engineering Department, Polytechnic University of Madrid, Jose Gutierrez Abascal 2, 28006 Madrid, Spain; jose.rios@upm.es; 3 German Research Center for Artificial Intelligence (DFKI), Trippstadter Straße 122, 67663 Kaiserslautern, Germany; klaus-dieter.althoff@dfki.de; 4 Institut für Informatik, University of Hildesheim, Universitätsplatz 1, 31141 Hildesheim, Germany

**Keywords:** product lifecycle management (PLM), manufacturing problem solving (MPS), fault diagnosis, smart factory, process failure mode and effect analysis (PFMEA), case-based reasoning (CBR)

## Abstract

Fault diagnosis presents a considerable difficulty to human operators in supervisory control of manufacturing systems. Implementing Internet of Things (IoT) technologies in existing manufacturing facilities implies an investment, since it requires upgrading them with sensors, connectivity capabilities, and IoT software platforms. Aligned with the technological vision of Industry 4.0 and based on currently existing information databases in the industry, this work proposes a lower-investment alternative solution for fault diagnosis and problem solving. This paper presents the details of the information and communication models of an application prototype oriented to production. It aims at assisting shop-floor actors during a Manufacturing Problem Solving (MPS) process. It captures and shares knowledge, taking existing Process Failure Mode and Effect Analysis (PFMEA) documents as an initial source of information related to potential manufacturing problems. It uses a Product Lifecycle Management (PLM) system as source of manufacturing context information related to the problems under investigation and integrates Case-Based Reasoning (CBR) technology to provide information about similar manufacturing problems.

## 1. Introduction

A manufacturing failure is an event in which some part of a manufacturing system does not perform according to its operational specifications and therefore it occurs in a specific manufacturing context. Due to such a failure, production is disturbed to a certain extent. The consequence is that production targets may not be achieved. The gap between the resulting production state and the intended production state is thus a problem. A Manufacturing Problem Solving (MPS) procedure starts when such a situation arises. The first step in an MPS procedure is the fault diagnosis, which presents considerable difficulty to human operators in supervisory control of manufacturing systems [1,2]. Providing support to these operators, by means of knowledge-based software applications, has been identified as essential to achieve good results from the solving procedure [3,4,5].

The characterization of the manufacturing context, where a failure might occur, can be set by means of processes, process steps, machines, tooling, process parameters, and product manufacturing features. Therefore, the context of the failure can be described by means of Process-Product-Resource (PPR) data. When looking at data, historical failure data could be used to assist in the MPS process. The use of such historical data would require identifying similar failures, which, in turn, requires making use of manufacturing context data. Product Lifecycle Management (PLM) systems are considered the main source of PPR digital information, and therefore their use would facilitate any attempt to develop a knowledge-based solution to support an MPS process [5,6]. PLM systems are one of the main components of the digital manufacturing and Industry 4.0 strategy [7,8].

The technological vision of a Smart Factory [9,10] is also included in the strategy Industry 4.0 [8], and eventually aims to develop a factory model that is context-aware and assists people and machines in the execution of their tasks. Fault diagnosis can be considered as one of these tasks to be supported.

A typical approach to a Smart Factory [9] is the deployment of smart artifacts, along the value streams, that are able to collect a large quantity of data from their environment and to communicate with each other (Internet of Things) [8]. This approach implies large investments, which prevents some companies from taking their first steps into Industry 4.0 [11,12]. This work presents an approach that aims to contribute to the technological vision of a Smart Factory but observing the constraints of being based on existing facilities, existing company data repositories, and with low investment.

The proposed approach is a knowledge-based development that integrates two main types of software applications: PLM and Case-Based Reasoning (CBR) [5]. The developed prototype assists human operators during an MPS process. It guides the user through the MPS activity based on the problem-solving method 8D [13]. The user introduces a query describing a problem that occurs in a production line, and the developed prototype software proposes several potential causes to be checked in the line. Based on the user feedback related to these first proposed causes, the system will propose the next lower level of causes. The process is repeated until the root cause of the problem is identified. More detailed information can be found in Camarillo et al. [5].

As sources of information, the prototype integrates two repositories. First, the existing company Process Failure Mode and Effect Analysis (PFMEA) records [14] are stored in the case base of the Case-Based Reasoning (CBR) system [15]. Second, the company’s Product Lifecycle Management (PLM) repository contains the PPR data. The Case-Based Reasoning (CBR) system is used to search for and retrieve information concerning similar problems. The similarity determination between the current problem and the problems contained in the case base depends on the PPR data stored in the PLM repository. The architecture of the system is based on SEASALT (Shared Experience using an Agent-based System Architecture LayouT) [16], which is a multi-case-based, domain-independent reasoning architecture for extracting, analyzing, sharing, and providing experiences. Camarillo et al. [17,18] show a description of a first prototype and initial results. Camarillo et al. [5] shows the MPS process model, the architecture of the developed prototype, the main ontology to support the MPS process, and the validation of the prototype. This paper provides an insight into the information and communication models of the developed prototype.

The next sections of this paper are structured as follows. Section 2 contains a review of research works with similar approaches. Section 3 and Section 4 discuss the created information and communication models, which are implemented in the developed prototype. Section 5 describes the configuration of the developed system. Section 6 provides a brief description of an application case. The paper ends with the conclusions.

## 2. Related Works

The developed approach is grounded on three basic models: an MPS process model, an MPS knowledge representation model, and an MPS system architecture model [5].

**Figure 1:**
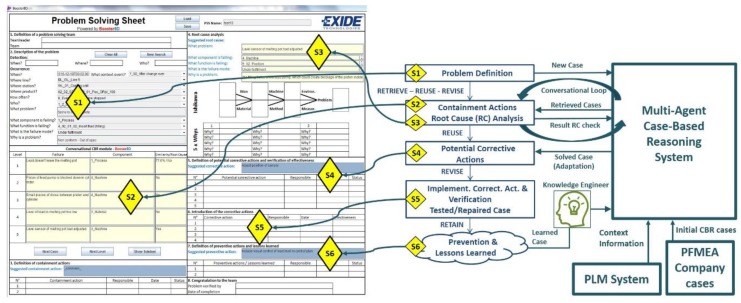
Process model of knowledge-based system. PLM, Product Lifecycle Management; PFMEA, Process Failure Mode and Effect Analysis; CBR, case-based reasoning.

The MPS process model defines the steps to be taken by the user to solve a problem with the support of the proposed system. This process model basically follows the steps defined in the 8D method [13] and specifies the kind of interaction expected at each step between the user and the system. The left side of Figure 1 shows the developed Graphical User Interface (GUI), the center part of Figure 1 shows the main steps of the MPS process, and the right side of Figure 1 shows the main systems of the developed prototype. The main steps of the MPS process are explained next and their links with the developed GUI are shown in Figure 1.

1. The user inputs a basic description of a manufacturing problem into the system (S1).
2. Based on the user input, the system searches and collects context information related to the problem from the PLM system repository. The result is shown to the user (S1).
3. Combining the input from the user and the data collected from the PLM repository, the system creates a global query to search for possible solutions (S2).
4. The system distributes the global query among its agents [16] that look for the most similar proposals out of their own case bases by applying CBR. The 10 most similar proposals coming from the agents are presented to the user. Initially, only the proposed containment actions and problem causes are displayed (S2).
5. The user must check the proposed failure modes or causes at the manufacturing location where the problem was identified and give feedback to the system. At this stage, the user may decide to refine his problem formulation and then go back to Step 1 (S2).
6. Once the possible root causes are identified, the system provides the related proposals for corrective and preventive actions (S3).
7. As part of the lessons learned step, the user gives feedback to be analyzed by a Knowledge Engineer. When appropriate, the Knowledge Engineer will update the CBR subsystems to extend the case bases (S4, S5, S6).

**Figure 2:**
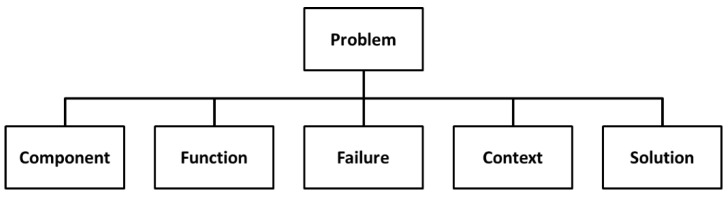
Manufacturing Problem Solving (MPS) top-level Ontology.

The MPS knowledge representation model is based on an ontology that allows for the representation of any knowledge related to the MPS process [5]. It comprises the following main concepts: Problem, Component, Function, Failure, Context, and Solution (Figure 2). The relations among these six concepts, their associated taxonomies, and their parameters have been designed to fulfil several constraints: support a generic definition of a manufacturing process and its location, be compatible with the information structure of the PFMEA method, comprise concepts to describe different aspects of a manufacturing problem, and to allow case similarity determination.

The proposed ontology defines the concept “Problem” similarly as in FMEA (Failure Mode and Effect Analysis) [14], where a component performs a function, and the latter fails in a defined mode. Component, Function, and Failure form a unique trio. The concept “Component” is subdivided into six subtypes: Process, Man, Machine, Material, Method, and Environment. The concept “Context” allows for representing the setting of a problem, is subdivided into seven different types of contexts: Material, Process, Machine, Event, Method, Man, and Environment, and has an associated taxonomy represented through the relationship type “is part of” pointing to itself. Each subtype of Context has different types of attributes to specify each type of technical information in the context (e.g., pressures, temperatures, and dimensions). These attributes are used in the configuration of the PLM system to store PPR information explicitly associated with the problem. More detailed information about this ontology can be found in Camarillo et al. [5].

The proposed MPS system architecture model is based on SEASALT (Shared Experience using an Agent-based System Architecture LayouT) [16]. The developed architecture supports the deployment of the different agents across different manufacturing plants of a company. Within each plant, agents can be deployed across the areas with different manufacturing processes. In this way, each topic agent, hosted in a specific manufacturing process of a specific manufacturing plant, will be able to collect and to store knowledge related to its own area, becoming an expert of its process and plant. By means of a coordinator agent, a topic agent can communicate and interchange information with all the other topic agents hosted in different processes and/or plants through the company’s intranet. Each topic agent has its own case base and uses CBR technology to find the most similar cases related to a user query. This information exchange supports the MPS process by providing the user with solutions for the most similar failures stored in any topic agent of the architecture [5].

In the literature review, several relevant works developed by other researchers were identified. Firstly, in relation to the modeling of PFMEA concepts. Dittmann et al. [19] presented an ontology to support FMEA concepts. The information model proposed in this work enhanced that ontology mainly by adding the concepts of Problem and Context [5]. In relation to the use of a PLM repository, Bertin et at. [6] propose, as part of a Lessons Learned System (LLS), the use of a PLM system as the central repository of data, but they put the focus on the Engineering Change Request (ECR) process of the company, whereas this work focuses on problem solving at production lines.

The work of Yang et al. [1] presents a fault diagnosis system for software intensive manufacturing systems and processes. They also profit from the stored information in the FMEA documents of the company and use CBR as an Artificial Intelligence (AI) tool. Nevertheless, they propose a second AI technology, deep-level Bayesian diagnosis network, to be used in cases of dynamic multi-fault diagnosis with uncertainty. The approach presented in this paper shares with them the use of FMEA and CBR but remains at a simpler AI level. However, the application scope of this work considers the sharing of knowledge among different manufacturing processes and plants (represented by topic agents). Also, contrary to the single-diagnosis suggestion proposed by Yang et al. [1], this proposed system uses an MPS method to guide the user step-by-step through the resolution of problems, which allows multiple cycles of problem redefinition, and that is fundamental when addressing very complex problems.

Finally, two relevant research works were identified in the field of fault diagnosis in aircraft maintenance: Chiu et al. [20] and Reus et al. [21]. Chiu et al. [20] propose the use of CBR together with genetic algorithms to enhance dynamic weighting and the design of non-similarity functions. With this approach, the proposed CBR system is able to achieve superior learning performance. As in the previous case, the approach presented in this paper remains at a simpler AI level, but proposes knowledge sharing among different MPS units. Reus et al. [21], as in this work, propose the use of SEASALT as a multi-agent architecture to share knowledge among multiple units. Nevertheless, the use of extended context-related information to enrich the similarity calculation is not addressed. Therefore, the link to a PLM system to enrich the similarity calculation and the search for solutions is outside its scope.

The next section introduces the developed information models and their link to the data sources, with special focus on the data related to PFMEA and the PPR concepts to be supported by the PLM system repository.

## 3. Information Models and Sources of Data

**Figure 3:**
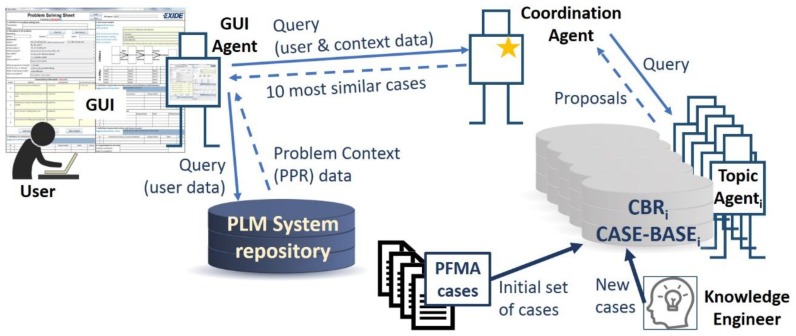
Top-level view of the proposed system data sources. GUI, graphical user interface; PPR, Process-Product-Resource; PFMA, Process Failure Mode Analysis.

The proposed knowledge-based system uses data from four different sources (Figure 3):

- User query: through the developed Graphical User Interface (GUI), the user introduces a rough definition of the problem that occurs in the production facility. Taking the Kepner–Tregoe method [22] as a reference, the user should provide an answer to the questions ‘What?’ (a brief description of the problem), ‘When?’ (date and time), ‘How often?’ (frequency), ‘Where?’ (this question is divided into three different attributes related to the line and station where the problem happens and the product that is being produced), ‘Who?’ (operator name), and ‘Why?’ (a brief description of why it is a problem).
- Problem context: based on the user query, the system receives from the PLM system repository multiple details about the associated context of the problem.
- Cases from PFMEA: the system needs an initial set of cases (i.e., problems previously solved), stored in the case base of the CBR system, in order to propose solutions to the user. This work proposes the use of the company’s available PFMEA documents to generate the initial case base.
- Knowledge engineer: an assigned employee in the company will analyze continuously the problems solved with the system and will create new cases to be stored in the case base (i.e., the reusing of created knowledge).

Figure 3 shows the top-level view of the knowledge-based system. The user manages the system through the GUI. The GUI represents a Problem-Solving Sheet (PSS) divided into the corresponding areas of the 8D method. The user inputs the description of the problem through the GUI as described at the beginning of this section. Based on the input description, a GUI agent will send a query to the PLM system to receive the contextual information related to the data input by the user. Then, a comprehensive query, comprising both the user’s input data plus the retrieved context data, is sent to the CBR system (Coordination Agent). The Coordination Agent will need to collect a first set of possible solved cases. The Coordination Agent communicates with the different topic agents to request proposals for similar problems. The case base of each topic agent needs to be populated with an initial set of cases (i.e., already solved problems). To do so, the company’s PFMEAs are taken as the initial set of cases. The case base will be continuously extended with new solved problems. A solved problem is analyzed by an expert to decide whether it is included as a new case in the CBR case bases or not. A deeper insight to this process is given in the next sections.

The next subsections focus on the developed information models used to collect cases from PFMEA and to collect context information from the PLM system.

### 3.1. PFMEA Data

**Figure 4:**
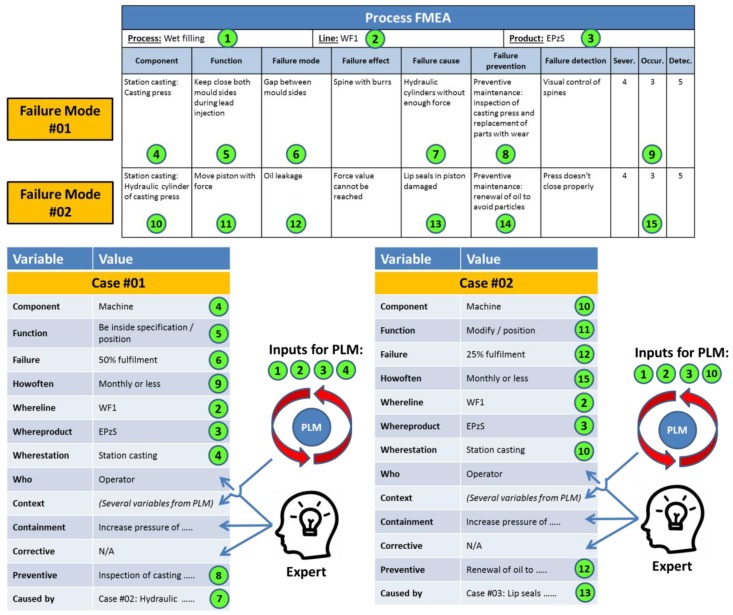
Information match between PFMEA and the knowledge model.

The initial source of cases for the knowledge-based system is the PFMEA documentation of the company. Figure 4 shows the information mapping between PFMEA and the knowledge model of the system [5]. As is shown in this figure, the PFMEA does not contain all the needed information to match totally the knowledge model of the system. Thus, the support of experts and the PLM system of the company are needed to translate the identified failures from the PFMEA into useful cases for the knowledge-based system. This situation derives from the fact that a PFMEA document represents a detailed analysis of all possible modes of failure associated with a specific production process [14]. However, the PFMEA document does not contain explicit technical information about the context of the process under analysis, and that information needs to be found in other documents associated with the PFMEA. In the proposed knowledge-based system, the created cases will be definitely detached from their original PFMEA documents, and the context information will have to be extracted from the PLM system of the company. This situation requires an expert to conduct a translation of failures into cases. A second issue to solve, in the translation activity, is that a PFMEA is a technique to prevent failures, but it is not designed to contain or to correct failures. Therefore, an expert has to fill these two fields, as well as define who is involved when the problem might happen (i.e., the parameter “Who”), a piece of information that is also not explicit in the PFMEA document.

The fields in the PFMEA matrix (Figure 4) marked with 1, 2, 3 (i.e., the process, production facility, and product under analysis), and 4 (i.e., the component where the potential failure is identified) are used as input for the PLM to search for and retrieve the context data. The fields 4, 5, and 6 represent the core of the problem definition (i.e., Component, Function, and Failure). Field 9 (i.e., occurrence) defines the frequency of the problem, field 7 the preventive action, and field 8 indicates which failure causes the problem. The same process is repeated systematically with every new failure.

### 3.2. PLM Information Model and Manufacturing Context Data

The PLM system has the function of providing the context data related to the user query. For this, the PLM system has to be customized to fit into the defined ontological model, so the requested context information can be used by the knowledge-based system. This customization has two main areas of activity:

- PLM systems are a repository of data related to the product lifecycle, but not all the data are stored necessarily as data records in the system repository (e.g., technical reports written using a word processor application could contain product or process parameters, but for the PLM repository the file is a black box, and it only knows the metadata associated with the “Technical Report” type of file). Therefore, the parameters defined by experts as relevant for the similarity calculation (i.e., the parameters that distinguish one process from another one) have to be declared as parameters in the PLM repository.
- The context information is retrieved based on the query defined by the user. As was mentioned in Section 3, the user inputs data about the affected production line, the station, the product, the user, and the date when the problem happened. Taking these data, the PLM system needs to access all the context-related data. This means that the system needs to move automatically from element to element, collecting as much data as possible. For this reason, specific relationships to link all the related items in the PLM system have to be created.

In the case study presented in this work, the selected company had no prior PLM system available. The software Aras Innovator (version 11.0) was selected as a PLM system and its installation and customization were part of the case study. On top of the two customization activities defined above, an experimental novel PLM information model was developed with the aim of accelerating the retrieval process of context data.

**Figure 5:**
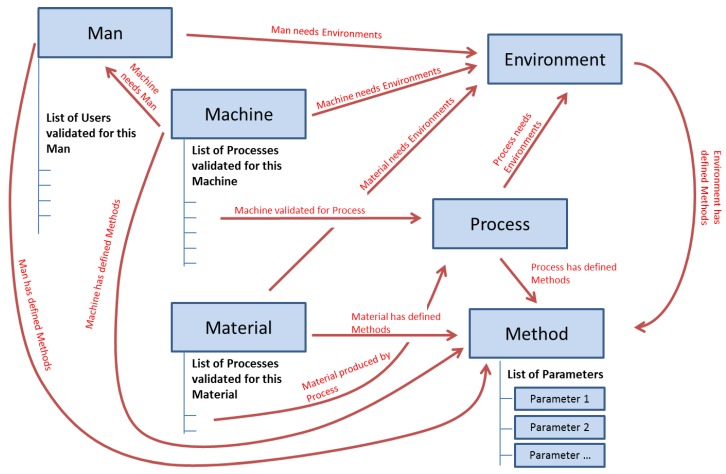
PLM structure of items.

The taxonomy of the class Context of the ontological model (Figure 5) has six main elements: Process, Machine, Material, Man, Method, and Environment. These six elements were declared as main elements in the PLM data structure (“Items” as per the standard terminology in Aras). Figure 5 shows these six elements with their relationships. These relationships will be the key to the process of information search as previously mentioned.

In the PFMEA methodology [14], on which the created ontology is partially based, the element Method represents the defined procedures or standards. Following this philosophy, the element Method contains the technical specification of each of the other component types in the PLM system.

A relevant issue in the management of technical information is that part of the information can be common for a whole family of components (e.g., a family of hex bolts with a specific diameter and thread where each one is distinguished from the others by length). In this sense, it is important to create a configuration in the PLM system that allows this type of information inheritance. This will ensure higher information consistency in the PLM system and a much easier update procedure. This was addressed by the creation of an additional element named “Parameter”. A Parameter is defined as the smallest stored unit of information in the PLM repository. It contains information about a single attribute, and it is made of the type of the attribute (e.g., pressure or temperature), its limit type (i.e., max, min, nominal, or not applicable), its value (either numerical or a selection from a set of possible values), and its measurement unit. Each instantiation of a Parameter can be shared by multiple instantiations of Method. For example, an instance of the element Process called “Casting Plate 90” will have a link to an instance of an element Method called “Casting Plate 90 method”, and this last one will contain a list of instances of the element Parameter, such as “Pressure/Max/10/bar” or “Temperature/Nom/300/°C”. These parameters can be also used by the Method instantiation “Casting Plate 125 method” when the parameter Pressure does not change with the size of the grid.

Thus, this independency of Parameter-related Method allows the same parameter to belong to different methods. This inheriting process should not be confused with the type of inheritance between classes in typical Object-Oriented Programming (OOP). In the OOP case a child class inherits the attributes of its parent class, but in the proposed structure both an attribute and the specific values of such an attribute are inherited from an instance of the parent class to the instance of the child class. With this idea, each of the component subclasses can have links to multiple Method elements containing several types of technical data related to the family of the component (e.g., a method to define the material and supplier applicable to all hex bolts made of stainless steel, a method to define the thread and diameter applicable to all hex bolts with a standard thread and a diameter of 5/8”, and a method to define the length applicable to a hex bolt with a length of 6”).

**Figure 6:**
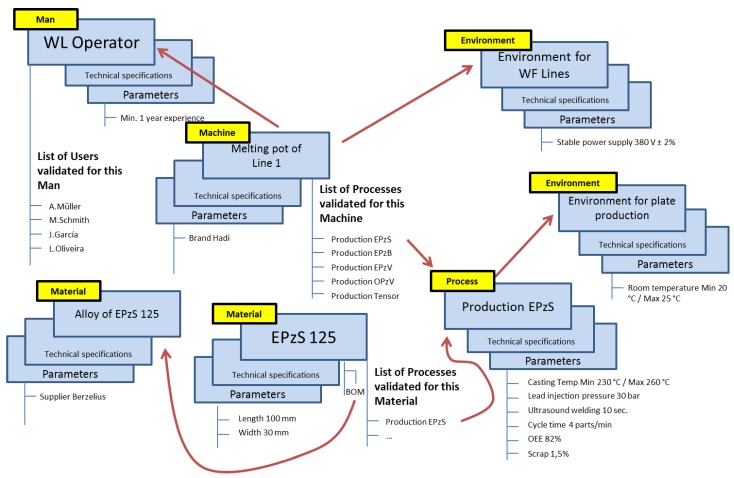
Example of the PLM structure of items (OEE: Overall Equipment Effectiveness).

The defined relationships and attributes for the PLM system are (Figure 6 shows an example):

- Machine attribute: each machine has a list of processes validated for it. This list can be shorter or longer depending on the complexity of the machine (e.g., pneumatic cylinder versus complete assembly line) and depending on the number of products that the machine can produce (each product can have specific processes).
- Machine to Man: a machine needs one or more human resources to control it. These human resources are part of “Man”, and they can be of different subtypes (e.g., operator, process engineer, quality inspector…).
- Machine to Environment: a machine works in a defined environment, which comprises specific conditions or requirements, for example, a limited range of room temperature, or a stable power supply.
- Machine to Method: a machine has defined methods, which contain the technical specification of each of the other element types.
- Material attribute: a material has a list of processes validated for producing it. As in the case of Machine, this list can be longer or shorter.
- Material to Environment: a material works in an environment.
- Material to Method: a material has defined methods.
- Process to Environment: a process works in an environment.
- Process to Method: a process has defined methods.
- Process to Machine and Material: a process has two indirect relationships with material and machine. A process is validated for a pair composed of a machine and a material. Each existing material has validated processes. Each machine has validated processes. The common process will define the manufacturing process of that material under production in that machine.
- Man attribute: a man has a list of users validated for it. It is important to highlight the difference between Man and User. Man represents a type of human resource (e.g., an operator), and User represents a specific person (e.g., Mr. Müller, who belongs to the type of Man operator).
- Man to Environment: a man works in an environment.
- Man to Method: a man has defined methods that can execute.
- Environment to Method: an environment has defined methods.
- Method to Parameter: a method has a list of parameters.

Finally, each of the explained elements will have a set of standard attributes that contains the basic data of the element. These attributes are:

- The Name of the element.
- The Element reference number, which is the identification number in the PLM system.
- The Revision level.
- The State (i.e., released or not).
- The Effective date from which the item is released.
- The Classification (i.e., the position of the component in the Context taxonomy).
- The Item Nature. This is an attribute to indicate whether the component is real or abstract. This attribute allows the user of the knowledge-based system, during the introduction of the problem description, to select a general family of products or lines instead of a specific one (e.g., “Casting Lines” versus “Casting Line 3”). Since the system searches for context data in the PLM repository, the PLM system must support also these general elements to be able to return data. Specific elements are tagged with “Real”, and general ones with “Abstract”.

The next section presents the communication models developed for this work. These models define the communication among the main four actors of the proposed system: user, agents, PLM system, and CBR system.

## 4. Communication Model

### 4.1. Communication among Agents and Users

**Figure 7:**
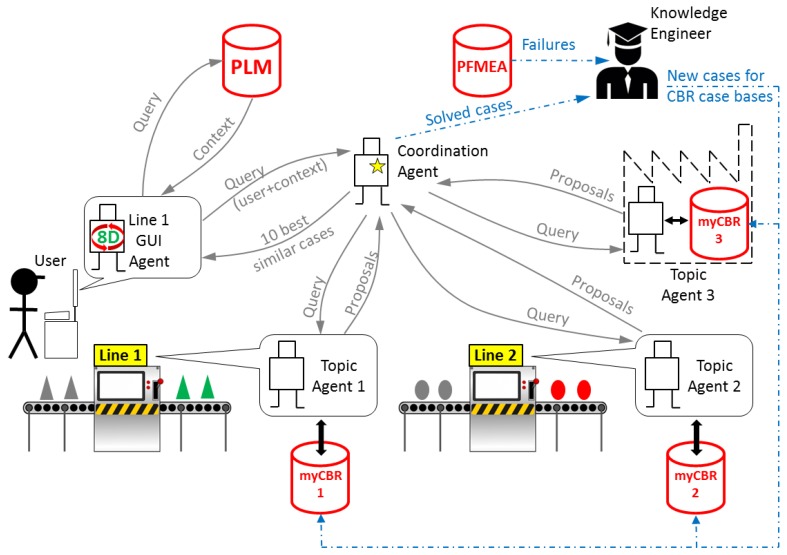
Communication model.

The communication model implemented in the developed system is a simplified version of SEASALT [16]. SEASALT is a multi-agent architecture made of three types of agents that communicate with each other to provide the user with solutions (Figure 7):

- GUI Agent: there are as many agents of this type as user access points in the network of the system. It manages the communication with the user, guiding him through the eight steps of the problem-solving method 8D. It manages also the communication with the PLM system (see Section 4.2) and the communication with the Coordination Agent.
- Topic Agent: there are as many agents of this type as production units in the network of the system. Each production plant should have at least one of these agents, and each production plant can have as many agents of this type as different production processes in the plant. Each Topic Agent has its own case base with solved cases of problems related to its production process, and it manages its own CBR engine to extract the most similar cases from its case base.
- Coordination Agent: there is a single agent of this type. It coordinates the communication among agents, and it is in charge of selecting the 10 most similar cases out of the multiple proposals coming from the topic agents.

The system has to be maintained by one or more knowledge engineers that are responsible for two main tasks:

- Translating failures stored in PFMEA documents into cases following the model presented in Section 3.1. This function could be automated to a large extent, but it is not part of the scope of this work, and it will be considered in future work.
- Analyzing problems that were solved with the system and rating their relevancy as a case to be added to the case base of a topic agent.

### 4.2. Communication with the PLM

The GUI Agent is responsible for communicating with Aras Innovator, the PLM system implemented in the case study company. The communication language of Aras Innovator is AML (Aras Markup Language). AML is an XML (Extensible Markup Language) dialect and markup language that drives the Aras Innovator server. Clients submit AML documents to the Aras Innovator server via HTTP (Hypertext Transfer Protocol), and the server returns an AML document containing the requested information.

The central element that builds Aras Innovator is the ItemType. An ItemType is a business object that represents the template or definition for the items that are created from it. In OOP (Object-Oriented Programming) terms, the ItemType is similar to the class definition, and the items that are created from it are the class instances or objects. Almost everything in Aras Innovator is defined through an ItemType. For instance, ItemType defines properties, forms, or views available for this item, its lifecycle, the work-flows associated with the item, permissions, relationships, server and client methods and events to run on the item, etc. ItemType is designed to hold as little information as a name, or as much information as required for the most complex business objects.

**Figure 8:**
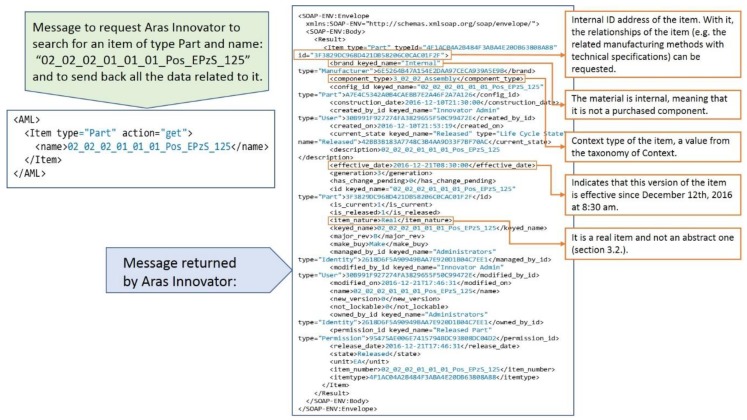
Example of communication with Aras Innovator.

The first step in any retrieval sequence is to get the ID (identification number) of the item that contains the requested information. Figure 8 shows an example of a message requesting Aras Innovator to search for an item of type Part (i.e., any mechanical design) and to send back all the data related to it and the returned message from Aras Innovator.

A very important point in the retrieval process from the PLM system is that the extracted context information should be that which was released and effective when the problem happened. The date is the parameter “When” in the user query (see Section 3). For example, the user may investigate a quality claim about a product that was created several months ago. In this case, the current version of context information in the PLM repository might not be relevant, because between the current date and the date when the problem happened several items could have been changed (e.g., new suppliers, changes in the design, or improvements in the processes). Aras Innovator returns by default the item released at the time of the request. Nevertheless, in case the release date is later than the date of the problem, there are functions that return all the previously released versions of an item.

Once the ID of an item is known, a message can be sent to the PLM system to request all its relationships. In the next example, all the manufacturing methods related to an item are requested.

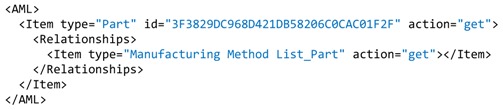



Following the relationships among elements defined in the PLM information model (Section 3.2), and with the AML queries presented above, it is possible to extract all the context information of a problem from the limited amount of information introduced by the user in the query (i.e., ‘What?’, ‘When?’, ‘How often?’, ‘Where?’, ‘Who?’, and ‘Why?’).

### 4.3. Communication with the CBR System

Section 4.1 described how the Topic Agent hosts the CBR system. For this case study, the open source system myCBR was selected [18]. Since both myCBR and the multi-agent architecture (on which the MPS system is built) are developed in Java, the communication between both systems is done directly through internal Java communication. The classes of both myCBR and the agent are instantiated in the same source code, and they can interchange information through their public functions.

The Topic Agent goes through the following communication steps to retrieve from its case base a set of similar cases related the user query:

- Sending an order to myCBR to load the CBR project of the production area where the agent is located. This project contains the information model to describe problems (based on the top-level ontology presented in Section 2), the defined rules to calculate similarity, and the name of the case base where similar cases will be searched for. These concepts will be explained with more detail in Section 5.
- Sending an order to myCBR to obtain the main concept of the information model of the project that was opened in the step before. In this case, this is “Problem”, the upper element of the MPS ontology (see Figure 1).
- Sending an order to myCBR to open the case base associated with the open project.
- Sending the retrieval parameters, which are the retrieval method and the problem attributes that are inside of the messaged received from the Coordination Agent.
- Launching the retrieval process.
- Receiving the 10 most similar proposals from of the open project.
- The proposals are sent to the Coordination Agent.

Once the way in which the CBR system receives a query and sends proposals is presented, the next section explains in detail how the CBR system manages the received data and how it calculates similarities in order to propose similar cases to the user.

## 5. CBR System Functioning Description

**Figure 9:**
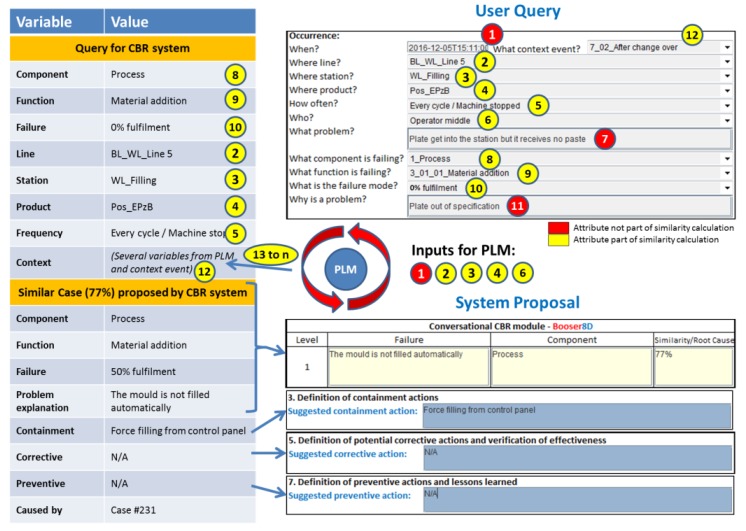
Information match between application user interface and the knowledge model.

**Figure 10:**
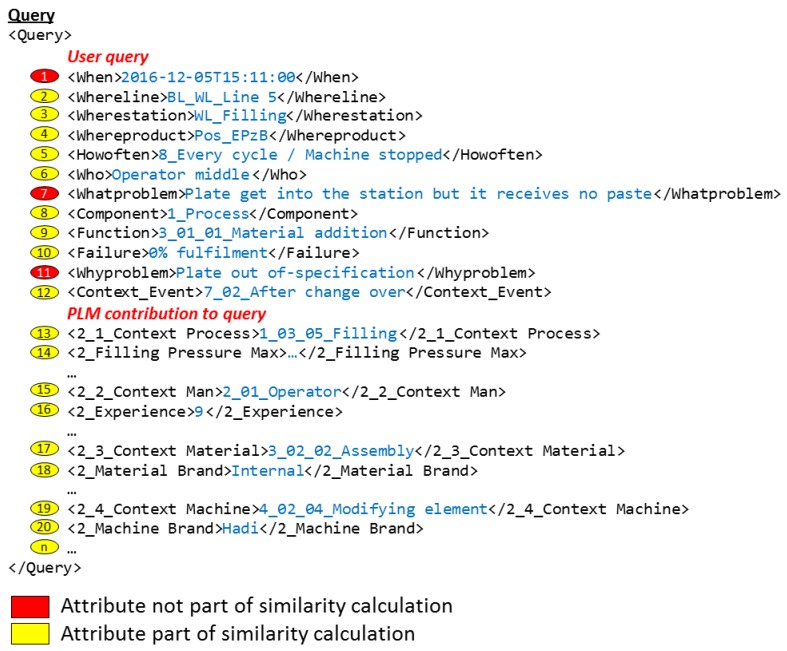
Example of query.

The CBR systems, which are hosted in each of the topic agents of the network, are responsible for seeking similar cases that could help the user to solve the current problem. For this function, they search in their own case base. The search is based on the information contained in the user query and the PLM context data received from the Coordination Agent. The user and PLM parameters are shown in the upper right corner of Figure 9. These parameters are presented also in Figure 10 with the format in which they are received by the topic agents. From the user inputs (numbers 1 to 12), some of the parameters (numbers 1, 2, 3, 4, and 6) are used in the request for context data in the PLM system as was explained in Section 3.2. The output of the PLM repository (number 13 to a previously undefined number “n” of attributes depending on each context case), together with some of the user query parameters (numbers 2, 3, 4, 5, 8, 9, 10, and 12), are used to create the query to be sent to the CBR system. This CBR query is shown in the upper left corner of Figure 9. Finally, there are some parameters in the user query (numbers 1, 7, and 11), shown in red in Figure 9, which are not used for the similarity calculation. The problem date (number 1) is used in the PLM system to find the released context information at the time that the problem happened. The parameters “What” and “Why” (numbers 7 and 11) are used by the knowledge engineer to understand better the solved problem, and to make a decision on whether the case will be included in the case base as a new case or not (see Section 4.1).

The parameters delivered to the CBR system should match with the internal information model of myCBR [5], which is built on the general knowledge model of the system, and which is used to describe the reality under analysis. myCBR works with projects, which are the basic container of classes, attributes, a Similarity Measure Function (SMF), instances of the classes, and case bases. Each project can contain one or more classes, and each class contains attributes of different types. Each numerical attribute has to be configured with its range of valid values, and each taxonomy attribute type has to be configured with its list of valid values. Finally, each attribute receives an SMF.

**Figure 11:**
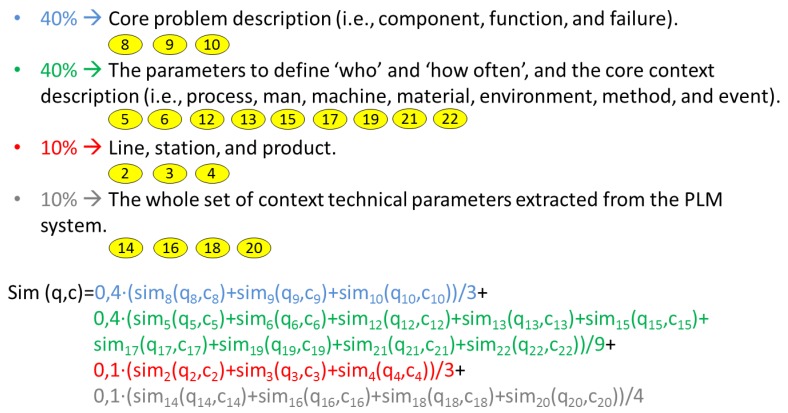
Weights for global similarity calculation.

SMFs are the functions associated with each attribute used by myCBR to calculate the final result, which will evaluate how similar is the received query in relation to each of the cases stored in the case base. Each attribute brings its own similarity contribution to the global similarity result, and the global similarity result is a pondered weight of all of them. Figure 11 shows the selected weights used in the case study of this work and the global formula applied in the project. Detailed information can be found in Camarillo et al. [5].

As previously mentioned, myCBR works with numerical and taxonomy attributes. Numerical attributes (e.g., numbers 2, 3, 4, 5, 14, or 16 in Figure 10) contain any value between a defined minimum and maximum. Taxonomy attributes (e.g., numbers 8, 9, 12, 13, or 20 in Figure 10) contain values from a specific list of valid values. The Similarity Measure Function (SMF) of numerical attributes calculates the relative distance between the value at query and case of this attribute. For example, in the attribute number 16, “experience”, if the query has the value 9 years, the case under analysis has the value 23 years, and the defined range for this attribute is from 0 to 45 years, the similarity contribution to the global value of this attribute would be:

sim(q,c) = 1 − |9 − 23|/|45 − 0| = 1 − 0.31 = 0.69.

**Figure 12:**
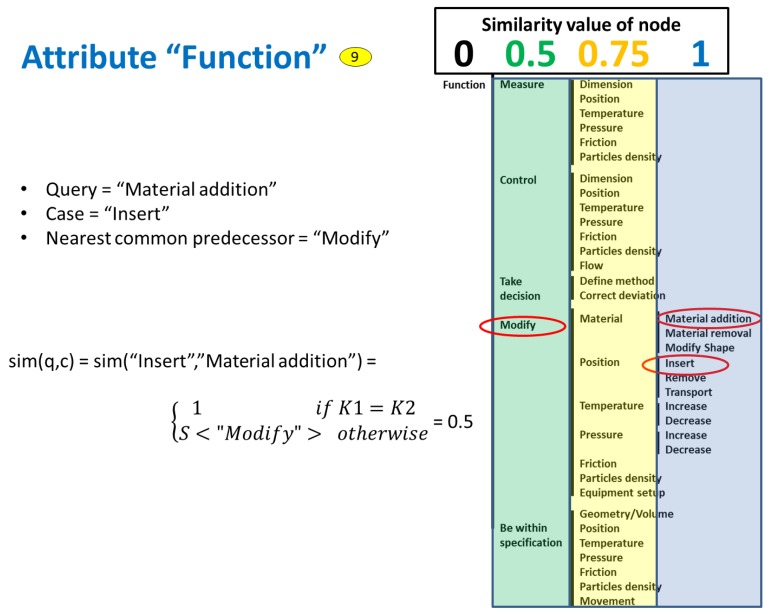
Example of individual similarity for taxonomy attributes.

The SMF of taxonomy attributes is calculated differently. The nodes of the taxonomy are associated with position factors, which are set based on experience and a trial-and-error process, until the results given by the system are satisfactory. The similarity value is calculated based on the value of the position factor of the common node related to both the query and the case. Figure 12 shows the example of the attribute “Function”, which is noted with number 9 in the figures above. In this case, the upper node (i.e., “Function”) was set with a position factor value of 0, the next one with value of 0.5, the following one with value of 0.75, and the lowest node with value of 1. To calculate the similarity, the SMF searches for the closest common node between query and case, which in this example is “Modify”. This node has a position factor of 0.5, and therefore the similarity result of the example is 0.5 as is shown in the figure.

A last relevant point related to similarity calculation is the range of supported manufacturing processes. In that sense, the developed system was designed to work for any kind of manufacturing process. Therefore, in the case of the context parameters, which are extracted from the PLM repository, a very wide range of possible parameter types should be expected. For some processes, a parameter of pressure might be relevant, but for other processes the relevant parameter could be a temperature. In the presented multi-agent architecture, each topic agent is specialized to the process where it is located. Therefore, when a topic agent receives a query, it will look for the parameters that it knows. Thus, all context parameters that are unknown by the topic agent will be ignored (i.e., no contribution in terms of similarity). It could also happen that not all of the parameters associated with its process are included in the query. In that case, the parameters that are not found will be assigned a null value, reducing the similarity result. In this way, cases from the same process will have always the highest similarity values followed by the cases from similar processes (i.e., processes with similar context parameters). The cases from very different processes will have the lowest similarity value.

Once the global similarity is calculated, the system displays the corresponding results. The bottom part of Figure 9 shows how the output in the system looks. In this case, the similarity value is 77%, and the proposal for the containment, corrective, and preventive actions is displayed to the user through the GUI.

The next section presents the details of the implementation of the developed system prototype in a case study company from the battery manufacturing sector.

## 6. Case Study in Battery Manufacturing

The developed system prototype was implemented as a case study in the company Exide Technologies, a global provider of stored electrical energy solutions (i.e., batteries and associated equipment and services) for transportation and industrial markets. Exide Technologies has several production plants running similar processes in Europe and the USA. For this reason, Exide could benefit from this research work.

For this case study, two plants of the company were selected: one located in Germany, and another one in Spain; both plants produce similar products with similar processes. They produce power batteries for the industrial market (i.e., forklifts or similar applications) with the process denominated Wet Filling to produce the positive plates. Gravity casting, with which the negative grids are produced, was also selected to test the performance of the system with a second manufacturing process.

The implementation of the case study can be divided in three phases: the definition of key PPR (Product-Process-Resource) attributes to ensure proper similarity calculation within the CBR systems, the collection of cases for the CBR case bases, and the test of the system on the shop floor.

As presented in Section 5, the similarity calculation is done based on a set of attributes that are selected out of the whole range of attributes that define the PPR reality of the case study. The selection criteria, and their individual weight in the similarity calculation, are related to the possible variation range of each attribute. For example, in a defined PPR scenario where the height of the product never changes across all existing part numbers, height would be a very bad candidate for similarity calculation, because it is not going to help to distinguish among cases. On the contrary, if the key differentiating characteristic of a range of products is the color, and there is a variety of colors, then color would be an excellent candidate for similarity calculation. To identify these key attributes for similarity, staff from production and engineering in the German plant of Exide were interviewed with a focus on the wet-filling process. The steps followed in these interviews were the identification of key PPR elements, the identification of the relationships among them, and finally the identification of their relevant attributes and their corresponding variability range. This information was used in the customization of both the PLM and CBR systems.

The next step in the case study was to collect enough cases to fill the CBR case bases of the topic agents. This activity was focused only on the wet filling production area of the German plant, leaving the Spanish plant for validation purposes. Four sources were used: the PFMEA of the wet-filling process, existing 8D reports coming from quality claims reported in the past, cases taken directly from the field in the German plant of the case study company, and some other cases from other manufacturing processes and companies, which helped also to test the capability and flexibility of the developed prototype. For the collection of cases directly on the shop floor, an unqualified individual without any kind of industrial background was engaged for two weeks, which helped to demonstrate that the proposed representation method for production problems is intuitive enough, and it is valid even for very unskilled operators. Table 1 shows a summary of all collected cases. In the table, the term “level” refers to any disruption that can be identified in production. The term “case” refers to a set of levels describing the whole chain of problems, from a visible one to the final root cause. The following example illustrates these concepts: a pump provides less pressure than defined, because the piston inside is worn out, because the lubrication oil has contamination particles, because the filter is broken. This example represents a single case with four levels.

For the last phase in the case study, several computers were installed in the selected production lines with access to the multi-agent architecture and to the PLM system. From these computers, the operators were able to access the problem-solving system to introduce their queries and receive recommendations from the system.

The group leaders of both plants were trained in the prototype system and they used it together with other operators to solve 10 problems occurring during their shifts. Since the cases in the prototype were expressed in English, the support of a translator was needed. The queries introduced by the users, the applicability of the results of the proposals coming out of the system, and the real solutions of the problems were all recorded for final analysis. The system was successfully tested, demonstrating the feasibility of the proposed approach [5].

## 7. Conclusions

This work has presented the details of the information and communication models of a developed system prototype to support a Manufacturing Problem Solving (MPS) process. The main contribution of the proposed system is that it integrates an MPS method with CBR on an agent-based distributed architecture and with a PLM system as a manufacturing context data repository. This novel approach had not been proposed in the reviewed literature.

The novelty from the modeling perspective resides in the information model created and implemented in a PLM system to facilitate the storage of and search for manufacturing context information that is used to calculate the similarity among production problems on the shop floor. The relationships among items in the system, and variables for the kind of explicit information related to each item, have been designed to facilitate the searching process associated with an MPS process, where the focus is placed on collecting as much contextual information as possible concerning the problem under investigation.

The approach is also an example of a low investment proposal that can be included in the conceptual frame of the technological vision Industry 4.0. Even if this case study is far from all advanced features envisaged today in the Smart Factory concept, it can be understood as a first small step to motivate some companies to start taking steps into Industry 4.0.

## Figures and Tables

**Table 1:** Case base of prototype application.

	Cases	Levels
Wet filling shop floor (German plant)	31	81
8D reports: Wet filling (German plant)	3	13
PFMEA: Wet filling (German plant)	16	60
Other processes/Other companies	16	72
**TOTAL**	**72**	**226**
